# Anomalous diffusion and asymmetric tempering memory in neutrophil chemotaxis

**DOI:** 10.1371/journal.pcbi.1010089

**Published:** 2022-05-18

**Authors:** Peter Dieterich, Otto Lindemann, Mats Leif Moskopp, Sebastien Tauzin, Anna Huttenlocher, Rainer Klages, Aleksei Chechkin, Albrecht Schwab

**Affiliations:** 1 Institut für Physiologie, TU Dresden, Dresden, Germany; 2 Institut für Physiologie II, Westfälische Wilhelms-Universität Münster, Münster, Germany; 3 Klinik für Neurochirurgie, Vivantes Klinikum im Friedrichshain, Berlin, Germany; 4 Department of Biology, Utah Valley University, Orem, Utah, United States of America; 5 Huttenlocher Lab, Department of Medical Microbiology, University of Wisconsin-Madison, Madison, Wisconsin, United States of America; 6 School of Mathematical Sciences, Queen Mary University of London, London, United Kingdom; 7 Max Planck Institut für Physik komplexer Systeme, Dresden, Germany; 8 Institute of Physics and Astronomy, University of Potsdam, Potsdam-Golm, Germany; 9 Faculty of Pure and Applied Mathematics, Hugo Steinhaus Center, Wrocław University of Science and Technology, Wrocław, Poland; 10 Institute for Theoretical Physics, NSC KIPT, Kharkov, Ukraine; National Institutes of Health, UNITED STATES

## Abstract

The motility of neutrophils and their ability to sense and to react to chemoattractants in their environment are of central importance for the innate immunity. Neutrophils are guided towards sites of inflammation following the activation of G-protein coupled chemoattractant receptors such as CXCR2 whose signaling strongly depends on the activity of Ca^2+^ permeable TRPC6 channels. It is the aim of this study to analyze data sets obtained in vitro (murine neutrophils) and in vivo (zebrafish neutrophils) with a stochastic mathematical model to gain deeper insight into the underlying mechanisms. The model is based on the analysis of trajectories of individual neutrophils. Bayesian data analysis, including the covariances of positions for fractional Brownian motion as well as for exponentially and power-law tempered model variants, allows the estimation of parameters and model selection. Our model-based analysis reveals that wildtype neutrophils show pure superdiffusive fractional Brownian motion. This so-called anomalous dynamics is characterized by temporal long-range correlations for the movement into the direction of the chemotactic CXCL1 gradient. Pure superdiffusion is absent vertically to this gradient. This points to an asymmetric ‘memory’ of the migratory machinery, which is found both in vitro and in vivo. CXCR2 blockade and TRPC6-knockout cause tempering of temporal correlations in the chemotactic gradient. This can be interpreted as a progressive loss of memory, which leads to a marked reduction of chemotaxis and search efficiency of neutrophils. In summary, our findings indicate that spatially differential regulation of anomalous dynamics appears to play a central role in guiding efficient chemotactic behavior.

## Author summary

Neutrophil granulocytes are essential for the first host defense. After leaving the blood circulation they migrate efficiently towards sites of inflammation. They are guided by chemoattractants released from cells within the inflammatory foci. On a cellular level, directional migration is a consequence of cellular front-rear asymmetry which is induced by the concentration gradient of the chemoattractants. The generation and maintenance of this asymmetry, however, is not yet fully understood. Here we analyzed the paths of chemotacting neutrophils with different stochastic models to gain further insight into the underlying mechanisms. Wildtype chemotacting neutrophils show an anomalous superdiffusive behavior. CXCR2 blockade and TRPC6-knockout cause the tempering of temporal correlations and a reduction of chemotaxis. Importantly, such tempering is found both in vitro and in vivo. These findings indicate that the maintenance of anomalous dynamics is crucial for chemotactic behavior and the search efficiency of neutrophils.

## Introduction

Being part of the innate immune system neutrophils play a crucial role during the first host defense. Their recruitment to a site of inflammation is an early event in the immune response and follows a well defined sequence of dynamical processes: rolling, adhesion, strengthening of adhesion, intravascular crawling and endothelial transmigration [1, 2]. After leaving the blood vessels neutrophils are guided by chemoattractant gradients to reach the site of inflammation [3]. On a molecular level chemotactic sensing involves the activation of G-protein coupled chemoattractant receptors such as CXCR2 whose activation trigger cytosolic signaling cascades underlying cell polarization in the chemotactic gradient and directed migration [4]. Chemotactic signaling frequently leads to a rise of the intracellular Ca^2+^ concentration by Ca^2+^ release from cytosolic stores and Ca^2+^ influx via Ca^2+^ permeable ion channels in the plasma membrane [5–8] such as members of the family of transient receptor potential (TRP) channels ([9–11] and [12] for a review). Impairing Ca^2+^ signaling interferes with neutrophil recruitment and chemotaxis [11]. We showed that TRPC6 channels are required for CXCR2-mediated chemotaxis of murine neutrophils [11]. Based on a conventional analysis of chemotaxis experiments that comprises speed, translocation and chemotaxis index, TRPC6^−/−^ neutrophils have a chemotaxis defect that appears quite similar to that caused by the inhibition of CXCR2 receptors in wildtype neutrophils. Thus, this standard analysis is not sensitive enough to distinguish between the different roles of CXCR2 receptors and TRPC6 channels or to propose a signaling hierarchy.

Different mathematical models have been suggested to explain the complex mechanisms underlying chemotaxis. At the level of a continuum description, examples include the polarized LEGI-BEN (local excitation global inhibition-biased excitable network) [13], excitable signal transduction networks [14], or pseudopod-centered models [15]. Chemotaxis was also analyzed within the context of Lévy walks for T cell migration in lymphoid tissue under in vivo conditions [16] or within the framework of Langevin equations for Dictyostelium [17]. Typical random walk models include temporal exponential correlations [18–21] and possible heterogeneities of cells [22]. Previously, we applied models of stochastic anomalous dynamics to analyze migration without chemotaxis and to quantitatively compare the phenotype of wildtype and mutant MDCK-F cells [23]. The latter are deficient for the sodium proton exchanger NHE1, a transport protein involved in persistent cell migration [23–25]. We discovered that cells are migrating superdiffusively. Superdiffusion of cell migration can be caused by a temporal memory effect. This in turn, is due to the temporal correlations of successive steps of cell migration. Thus, superdiffusively migrating cells maintain their direction of movement for longer time periods as those performing an uncorrelated random walk. We also found that this superdiffusive mode of migration is not affected by NHE1 deficiency. NHE1 rather stabilizes the polarization of migrating cells. Such behavior of polarization has later been modeled as ‘polarized excitable network’ producing persistent migration in the absence of chemotactic stimulation [26]. Thus, the model-based mathematical analysis has provided a much deeper insight into the mechanisms of cell migration without influences of chemotactic stimuli.

We therefore set out to develop an extended mathematical model that is based on stochastic dynamics of individual cell paths in order to analyse chemotaxis of neutrophil granulocytes in more detail. We used the same data set as in our previous work [11] and complemented it with data from in vivo experiments performed in zebrafish embryos [27]. Our results show that chemotaxis (in vitro and in vivo) leads to an anomalous dynamics with reduced tempering in the direction of the chemotactic gradient. Tempering can be interpreted as progressive loss of memory of the migration machinery. In addition, we find a much stronger effect of CXCR2 blockade than TRPC6-knockout on anomalous behavior which translates into different search efficiencies of the neutrophils. Thus, the model-based analysis of the chemotactic behavior of neutrophils yields a depth of insight on the molecular and systems levels that could not have been achieved with conventional data analysis.

## Results

### Overview of chemotaxis experiments

**Fig 1:**
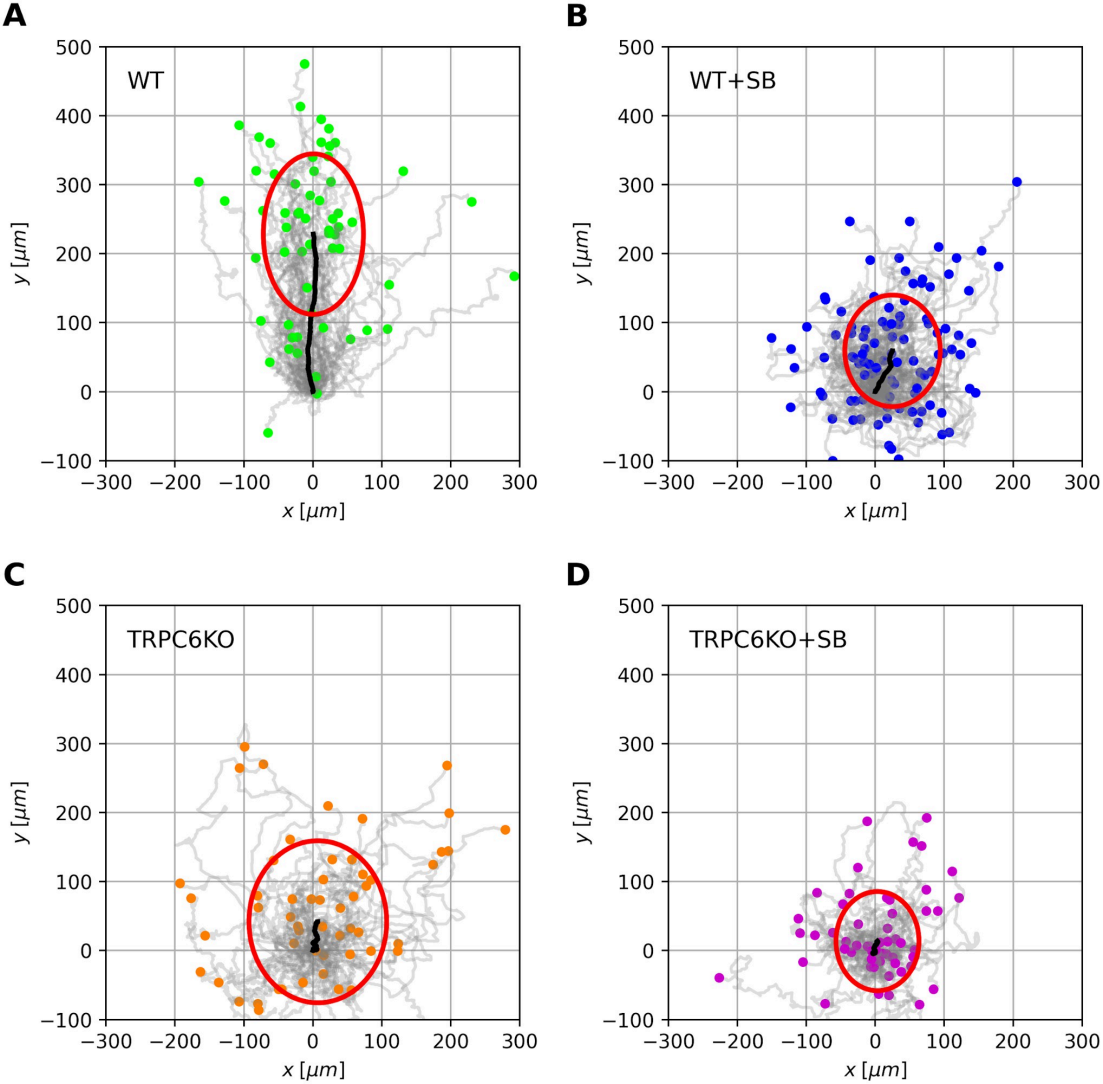
Overview of trajectories and mean drift behavior for murine neutrophils. (A)—(D) Paths of individual cells are normalized to a common starting point at the origin of the coordinate system. They are derived from different experimental conditions. Murine neutrophils were observed with a sampling interval of *dt* = 5 s over a time period of *T* = 30 min. Final cell positions are marked by colored circles (A-D). The black line indicates the mean position of the corresponding cell ensemble over the observation period *T*. The mean squared displacement of the cell ensemble after observation time *T* is represented as a red ellipse. The size of the ellipse indicates different propagation of cells in *x*- and *y*-direction. Murine neutrophils are chemotacting in a CLCX1 gradient along the positive *y*-axis. (A) Wildtype cells (WT). (B) Wildtype cells in the presence of the CXCR2 antagonist SB225002 (WT + SB). (C) TRPC6-knockout cells (TRPC6^−/−^). (D) TRCP6-knockout cells in the presence of CXCR2 antagonist SB225002 (TRPC6^−/−^ + SB).

Fig 1 summarizes the chemotaxis experiments with the trajectories of the murine neutrophil groups used in this study. Trajectories are normalized to common starting points with the coordinates (*x*, *y*) = (0, 0). Under control conditions wildtype neutrophils migrate efficiently along the CXCL1 (KC) gradient towards the positive *y*-axis (Fig 1A). This is indicated by the black line which depicts the mean position of the cell population during the course of the experiment. The black lines are much shorter in Fig 1B–1D because directed migration along the CXCL1 gradient is severely impaired by CXCR2 blockade (Fig 1B and 1D) and TRPC6 knock-out (Fig 1C and 1D).

The red ellipses represent the mean squared propagation of the cell positions in *x*- and *y*-direction with respect to their common center at the final time point of the experiments. The size of the ellipse is considerably smaller after CXCR2 blockade (Fig 1B). The asymmetry of the ellipse, which is particularly prominent for wildtype cells, indicates that murine neutrophils seem to migrate differently in *x*- and *y*-direction due to the influence of the chemotactic gradient. This asymmetry is lost when CRCX2 receptors are blocked or TRPC6 channels are absent.

**Fig 2:**
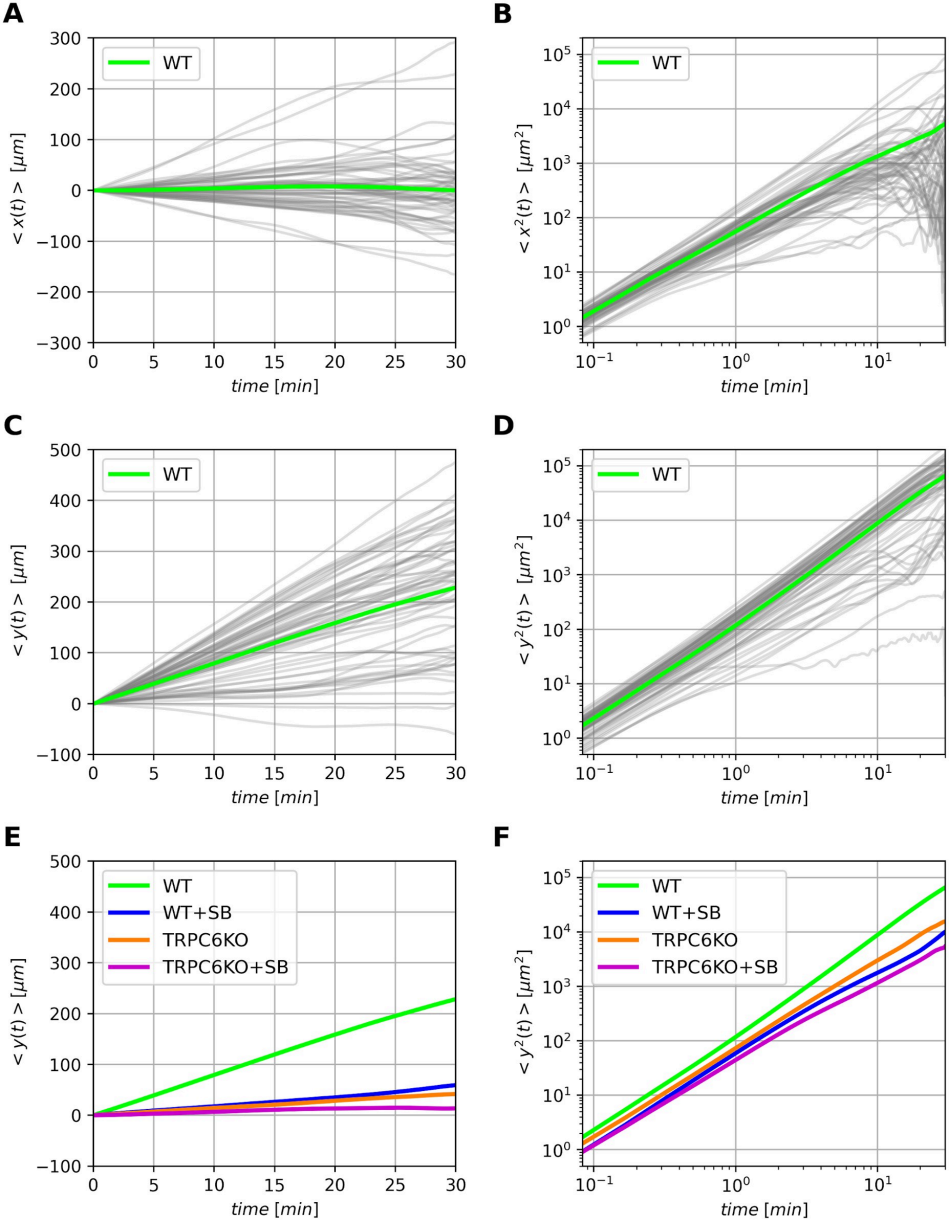
Overview of the first and second moments. (A), (B) and (C), (D) show the first and second moments of wildtype neutrophils with respect to *x*- and *y*-direction, respectively. Thin grey lines indicate time-averaged moments of single cells according to Eqs 16 and 17. The thick green lines show the time-ensemble-average of the corresponding moment according to Eqs 18 and 19. (E) compares the mean first moment of different experimental conditions with murine neutrophils. (F) shows the differences of the second moments, i.e. the mean squared displacement for murine neutrophils.

In order to further analyze the chemotactic behavior we calculated the first and second moments of displacements from the starting positions for individual neutrophils as a function of time under different conditions according to Eqs 16–19 (see Material and methods). The first moment characterizes directed motion (typically designated as drift), whereas the second moment contains additional information about the diffusive spreading of cells. The second moment is also called mean squared displacement. Fig 2A–2D show these moments for neutrophils from wildtype mice. Grey lines indicate the time-average results for each single cell (Eqs 16 and 17) and the green line shows the time- and ensemble-average of all cells as given by Eqs 18 and 19. As expected, there is a drift only in parallel to the chemotactic gradient (Fig 2C), which increases linearly in the direction of chemotaxis. It is absent perpendicular to the gradient (Fig 2A). Analogously we plotted the mean squared displacement in Fig 2B and 2D. Note that the second moment in the direction of chemotaxis is by more than one order of magnitude larger than in the perpendicular direction. This reflects the contribution of the drift. Fig 2E and 2F summarize the mean drift and mean squared displacement for all four experimental conditions studied in murine neutrophils. CXCR2 and TRPC6-knockout lead to an almost identical decrease of the mean drift velocity *v*_*d*_ given as slope of 〈*y*(*t*)〉. Combined CXCR2 inhibition and TRCP6-knockout cause a small additional reduction. Wildtype cells have a drift velocity of ∼ 7.4 *μ*m/min. Applying the CXCR2 inhibitor SB225002 reduces the drift to ∼ 25% of this control value. TRPC6-knockout cells also have a similarly diminished chemotatic drift, which is further reduced by blocking the CXCR2 receptor in these cells. TRPC6KO differentially affects drift and mean squared displacement: The drift is more strongly reduced by the TRPC6KO than the mean squared displacement. This is consistent with our previous observation [11] that TRPC6KO predominantly impairs the chemtotatic efficiency rather than the migration motor.

### Velocity autocorrelations of chemotacting neutrophils

**Fig 3:**
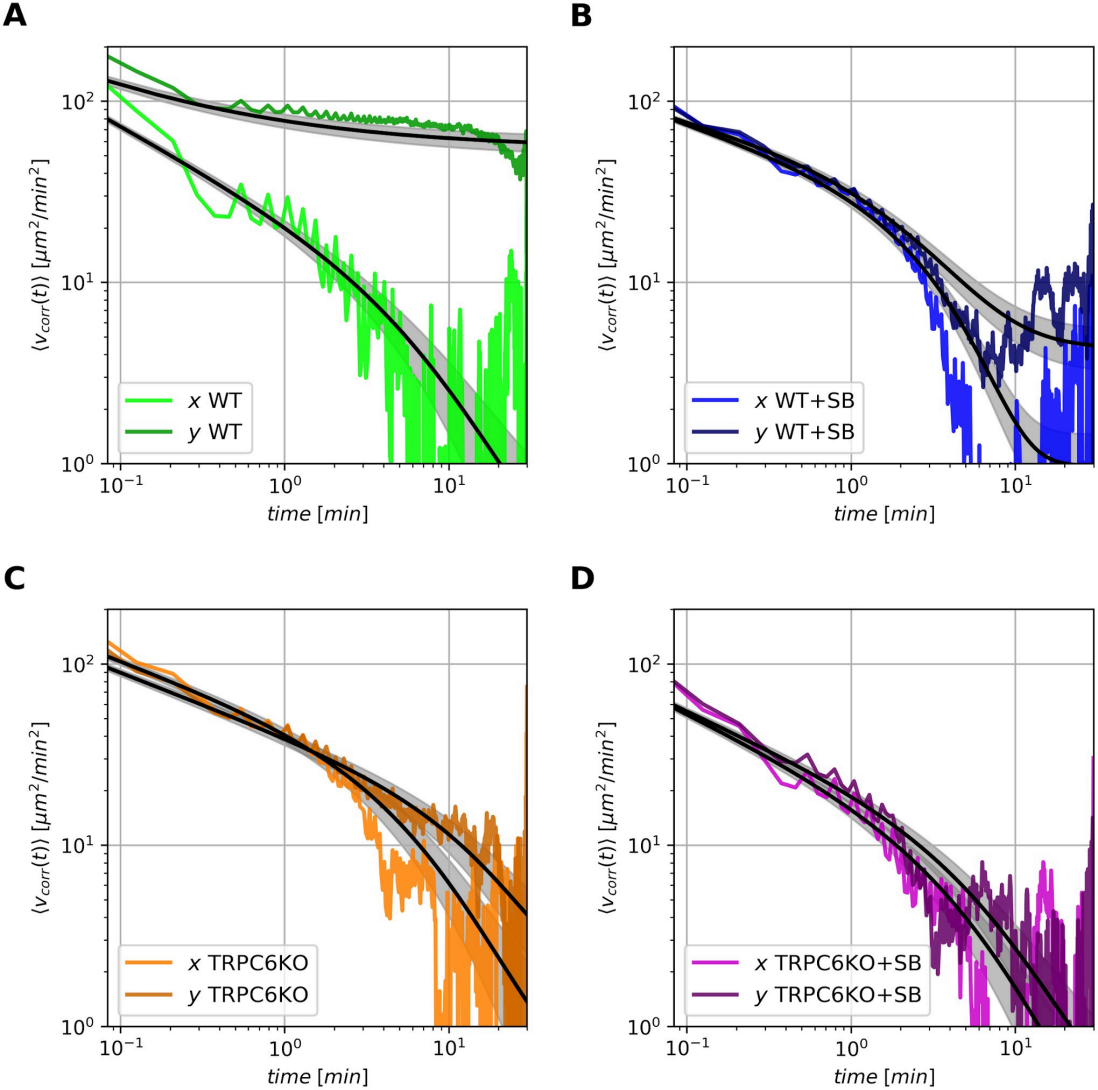
Velocity autocorrelation functions. The diagrams show the velocity autocorrelations in *x*- and *y*-direction. Thick colored lines indicate the corresponding time-ensemble-averaged velocity autocorrelation function 〈*v*_*corr*,*x*|*y*_(*t*)〉 as defined in Eq 22 with repect to *x*- and *y*-direction in bright and dark colors, respectively. The results of the best theoretical model are shown as black lines together with a grey area of uncertainty (± one standard deviation). (A) Murine wildtype neutrophils. (B) Wildtype neutrophils with CXCR2 blocker SB225002. (C) TRPC6^−/−^ neutrophils. (D) TRPC6^−/−^ neutrophils with CXCR2 blocker SB225002.

In order to analyze the migration behavior of the neutrophils in more detail we calculated the velocity autocorrelation functions based on Eqs 21 and 22. They provide information about the temporal memory of migrating neutrophils. Fig 3 shows the time-ensemble-averaged velocity autocorrelation functions 〈*v*_*corr*,*x*|*y*_(*t*)〉 for murine neutrophils. In general, these velocity autocorrelations slowly decay as shown in Fig 3 indicating a certain kind of temporal persistence which cannot be described by an uncorrelated random walk process. The finding of strong temporal autocorrelations is consistent with the model of Andrew and Insall [28] who proposed that lamellipodia preferentially grow from already existing protrusions. The experimental velocity correlation functions show typical noisy scatterings for longer times (*t* ≳ 10 min) thereby impeding a direct quantification of the decay as purely exponential or power-law. The velocity autocorrelation contains the diffusive and drift components of the stochastic process: Starting from 〈*v*(*t* = 0)^2^〉 at time *t* = 0 it decays towards the mean squared drift velocity $v_{d}^{2}$ for long times. In addition, we analyzed the velocity cross-correlations 〈*v*_*cross*,*xy*_(*t*)〉 according to Eqs 23 and 24 and did not find couplings of velocities between *x*- and *y*-direction (see S3 Data).

### Development of an optimal stochastic model for neutrophil chemotaxis

The migratory behavior of neutrophils in a chemotactic gradient as shown in Fig 1 is governed by two main processes: The first process refers to the directed migration of the cell population reflecting the effect of the external force induced by the chemotactic gradient. This external force can be assumed as constant since the mean position of cells along the *y*-axis increases linearly in time (Fig 2E). Diffusive spreading constitutes the second process. However, the temporal persistence of trajectories questions whether the migration process follows an uncorrelated random walk. The slow temporal decay of the velocity autocorrelation function as shown in Fig 3 clearly indicates temporal correlations. This is beyond the characteristics of ordinary Brownian motion, which is a totally uncorrelated process [29, 30]. Thus, these observations suggest to model cell migration as a correlated stochastic process, where the position of the cell (*x*, *y*) is influenced by an external constant force modeled by *v*_*d*_ (CXCL1 gradient) and an active stochastic force *ζ*_*x*|*y*_(*t*) generated by the cellular migration apparatus:

$$\begin{array}{c} \frac{dx(t)}{dt}=\zeta_{x}\left(t\right),\;\;\;\frac{dy(t)}{dt}=v_{d}+\zeta_{y}\left(t\right). \end{array}$$ (1)

This approach corresponds to an overdamped Langevin equation driven by (fractional) noise. Couplings of the motion in *x*- and *y*-direction can be neglected due to missing cross-correlations of velocities. As the experimental data of the velocity autocorrelation function do not allow a unique identification of its decay as exponential or power-law form, we tested the validity of three models (A—C) beyond the Ornstein-Uhlenbeck with exponential correlations (model D) [18–22]. These models consider different types of temporal correlations and tempering mechanisms. Tempering accelerates the decay of autocorrelations. Thereby, it may allow a transition from anomalous to normal dynamics. The models are defined in the following.

**Model A**: Pure fractional Brownian noise with correlations [30, 31]

$$\begin{array}{c} {\left\langle v^{2}\left(\tau \right)\right\rangle }_{A}={\left\langle \zeta \left(t\right)\zeta \left(t^{\prime }\right)\right\rangle }_{A}=\frac{D_{H}}{\Gamma (2H-1)}\;\tau^{2H-2} \end{array}$$ (2)

with the generalized diffusion coefficient *D*_*H*_ and the Hurst coefficient *H* (0 < *H* < 1). Γ represents the gamma function. Model A is characterized by a temporal power-law decay ∼ *τ*^2*H*−2^ with *τ* = |*t* − *t*′|. The model reduces to Brownian motion for *H* = 1/2 and shows superdiffusive behavior for *H* > 1/2. The special case *H* = 1/2 can be obtained from the asymptotic expansion of the gamma function $1/\Gamma \left(\epsilon \right)∼\epsilon +O\left(\epsilon^{2}\right)$ for *ϵ* → 0 [32] and a representation of the delta function $\delta (\tau )=\underset{\epsilon \to 0}{\text{lim}}\;\epsilon /2|\tau {|}^{\epsilon -1}$ [33]. With *ϵ* = 2*H* − 1 the limit of *ϵ* → 0 corresponds to *H* → 1/2. Thus, the correlation function reduces to 〈*v*^2^(*τ*)〉_*A*_ = 2 *D*_1/2_ *δ*(*τ*) for *H* = 1/2.

**Model B**: Fractional Brownian noise with exponential tempering [34]

$$\begin{array}{c} {\left\langle v^{2}\left(\tau \right)\right\rangle }_{B}={\left\langle \zeta \left(t\right)\zeta \left(t^{\prime }\right)\right\rangle }_{B}=\frac{D_{H}}{\Gamma (2H-1)}\;\tau^{2H-2}\;\text{exp}\left(-\tau /\tau^{*}\right) \end{array}$$ (3)

with tempering time τ*. The addition of the exponential term to model B causes an exponential loss of correlations for times > τ*. Model B reduces to model A for τ* → ∞.

**Model C**: Fractional Brownian noise with power-law tempering [34]

$$\begin{array}{c} {\left\langle v^{2}\left(\tau \right)\right\rangle }_{C}={\left\langle \zeta \left(t\right)\zeta \left(t^{\prime }\right)\right\rangle }_{C}=\frac{D_{H}}{\Gamma (2H-1)}\;\tau^{2H-2}\;{\left(1+\tau /\tau^{*}\right)}^{-\mu } \end{array}$$ (4)

with tempering time τ* and power-law tempering exponent *μ* ≥ 0. Model C is identical with model A for *μ* = 0 or τ* → ∞.

**Model D**: Ornstein-Uhlenbeck like processes are often used as reference processes to describe and quantify cell migration [18–22]. Thus, we also included this approach, which is characterized by an exponentially decreasing velocity autocorrelation function:

$$\begin{array}{c} {\left\langle v^{2}\left(\tau \right)\right\rangle }_{D}={\left\langle \zeta \left(t\right)\zeta \left(t^{\prime }\right)\right\rangle }_{D}=v_{m}^{2}\;\text{exp}\left(-\gamma \;\tau \right) \end{array}$$ (5)

Parameter $v_{m}^{2}$ denotes the mean squared velocity of cells and *γ* captures the decay of the exponential function with time-scale ∼ 1/*γ*.

Our models A—D are Gaussian processes that are fully characterized by their first and second moments. The first moments follow directly from Eq 1 and are identical for models A—D:

$$\begin{array}{c} {\left\langle x\left(t\right)\right\rangle }_{A|B|C|D}=0\;\;\;\;{\left\langle y\left(t\right)\right\rangle }_{A|B|C|D}=v_{d}\;t\;. \end{array}$$ (6)

The second moments given by the covariances of these processes can be obtained by integrating the velocity autocorrelations of Eqs 2–5 twice:

$$\begin{array}{c} {\left\langle x\left(t\right)x\left(t^{\prime }\right)\right\rangle }_{A|B|C|D}=\int_{0}^{t}dt_{1}\;\int_{0}^{t^{\prime }}dt_{2}\;{\left\langle \zeta \left(t_{1}\right)\zeta \left(t_{2}\right)\right\rangle }_{A|B|C|D}\;. \end{array}$$ (7)

They are different for each model:

$$\begin{array}{ccc} {\left\langle x\left(t\right)x\left(t^{\prime }\right)\right\rangle }_{A} & = & \frac{D_{H}}{\Gamma (2H+1)}\;[{\left|t\right|}^{2H}\;+|t^{\prime }{|}^{2H}\;-\;{\left|t-t^{\prime }\right|}^{2H}\;] \end{array}$$ (8)

$$\begin{array}{ccc} {\left\langle x\left(t\right)x\left(t^{\prime }\right)\right\rangle }_{B} & = & \frac{D_{H}}{\Gamma (2H+1)}\;[{\left|t\right|}^{2H}{\;}_{1}F_{1}\left(2H-1,2H+1;-\frac{\left|t\right|}{\tau^{*}}\right)\\ & & \;+\;|t^{\prime }{|}^{2H}{\;}_{1}F_{1}\left(2H-1,2H+1;-\frac{|t^{\prime }|}{\tau^{*}}\right)\\ & & \;-\;|t-t^{\prime }{|}^{2H}{\;}_{1}F_{1}\left(2H-1,2H+1;-\frac{|t-t^{\prime }|}{\tau^{*}}\right)] \end{array}$$ (9)

$$\begin{array}{ccc} {\left\langle x\left(t\right)x\left(t^{\prime }\right)\right\rangle }_{C} & = & \frac{D_{H}}{\Gamma (2H+1)}\;[{\left|t\right|}^{2H}{\;}_{2}F_{1}\left(\mu ,2H-1,2H+1;-\frac{\left|t\right|}{\tau^{*}}\right)\\ & & \;+\;|t^{\prime }{|}^{2H}{\;}_{2}F_{1}\left(\mu ,2H-1,2H+1;-\frac{|t^{\prime }|}{\tau^{*}}\right)\\ & & \;-\;|t-t^{\prime }{|}^{2H}{\;}_{2}F_{1}\left(\mu ,2H-1,2H+1;-\frac{|t-t^{\prime }|}{\tau^{*}}\right)] \end{array}$$ (10)

$$\begin{array}{ccc} {\left\langle x\left(t\right)x\left(t^{\prime }\right)\right\rangle }_{D} & = & \frac{v_{m}^{2}}{\gamma^{2}}\;[2\;\gamma \;t^{\prime }-1-\text{exp}(-\gamma \;|t-t^{\prime }\left|\right)+\text{exp}\left(-\gamma \;t\right)+\text{exp}\left(-\gamma \;t^{\prime }\right)\;]. \end{array}$$ (11)

_1_*F*_1_ and _2_*F*_1_ represent the confluent hypergeometric and the Gaussian hypergeometric function, respectively [32]. The *y*-direction contains additional terms $v_{d}^{2}\;t\;t^{\prime }$ due to the drift velocity *v*_*d*_, where model parameters (*H*, *D*_*H*_, τ*, *μ*) and $\left(\gamma ,v_{m}^{2}\right)$ can take different values with respect to *x*- and *y*-direction. The corresponding second moments follow directly from the covariances of Eqs 8–11 for *t* = *t*′ as

$$\begin{array}{ccc} msd_{x}{\left(t\right)}_{A} & = & \frac{2D_{H}t^{2H}}{\Gamma (2H+1)}\;, \end{array}$$ (12)

$$\begin{array}{ccc} msd_{x}{\left(t\right)}_{B} & = & \frac{2D_{H}t^{2H}}{\Gamma (2H+1)}{\;}_{1}F_{1}\left(2H-1,2H+1;-\frac{\left|t\right|}{\tau^{*}}\right), \end{array}$$ (13)

$$\begin{array}{ccc} msd_{x}{\left(t\right)}_{C} & = & \frac{2D_{H}t^{2H}}{\Gamma (2H+1)}{\;}_{2}F_{1}\left(\mu ,2H-1,2H+1;-\frac{\left|t\right|}{\tau^{*}}\right), \end{array}$$ (14)

$$\begin{array}{ccc} msd_{x}{\left(t\right)}_{D} & = & \frac{2\;v_{m}^{2}}{\gamma^{2}}\;(\gamma \;t-1+\text{exp}(-\gamma \;t))\;. \end{array}$$ (15)

Analogously to the covariances, the *msd* in *y*-direction *msd*_*y*_(*t*)_*A*|*B*|*C*|*D*_ is extended by the drift factor $v_{d}^{2}\;t^{2}$. In addition, the mean squared displacements for models A to C in Eqs 12–14 reduce to normal diffusion 2 *D*_1/2_ *t* for *H* → 1/2 as _1_*F*_1_(0, 2; *z*) = 1 and _2_*F*_1_(*μ*, 0, 2; *z*) = 1 [32]. The *msd*_*x*_(*t*)_*D*_ of the Ornstein-Uhlenbeck process in Eq 15 displays a ballistic phase $∼v_{m}^{2}\;t^{2}$ for small times *t* ≪ 1/*γ* and normal diffusion $∼2\;v_{m}^{2}/\gamma \;t$ for long times *t* ≫ 1/*γ*.

### Application of the model to experimental data

#### Bayesian data analysis

Bayesian data analysis [35, 36] is applied in order to link experimental data and the stochastic model for positions described in Eq 1. The Bayesian formalism including the covariances of the stochastic process (see Eqs 28 and 29) allows a logically consistent estimation of model parameters and their uncertainties. In addition, it provides the evidence for selecting the best model. Both calculations are based on the amount and quality of the experimental data. It is noteworthy, that the Bayesian formalism uses the positions of cell paths (raw experimental data) instead of performing a parameter fitting to the averaged mean squared displacement data. To avoid systematic errors due to pixelation of spatial coordinates, data were analyzed with *dt* = 10*s*, i.e. with every second data point.

#### Model classification

**Fig 4:**
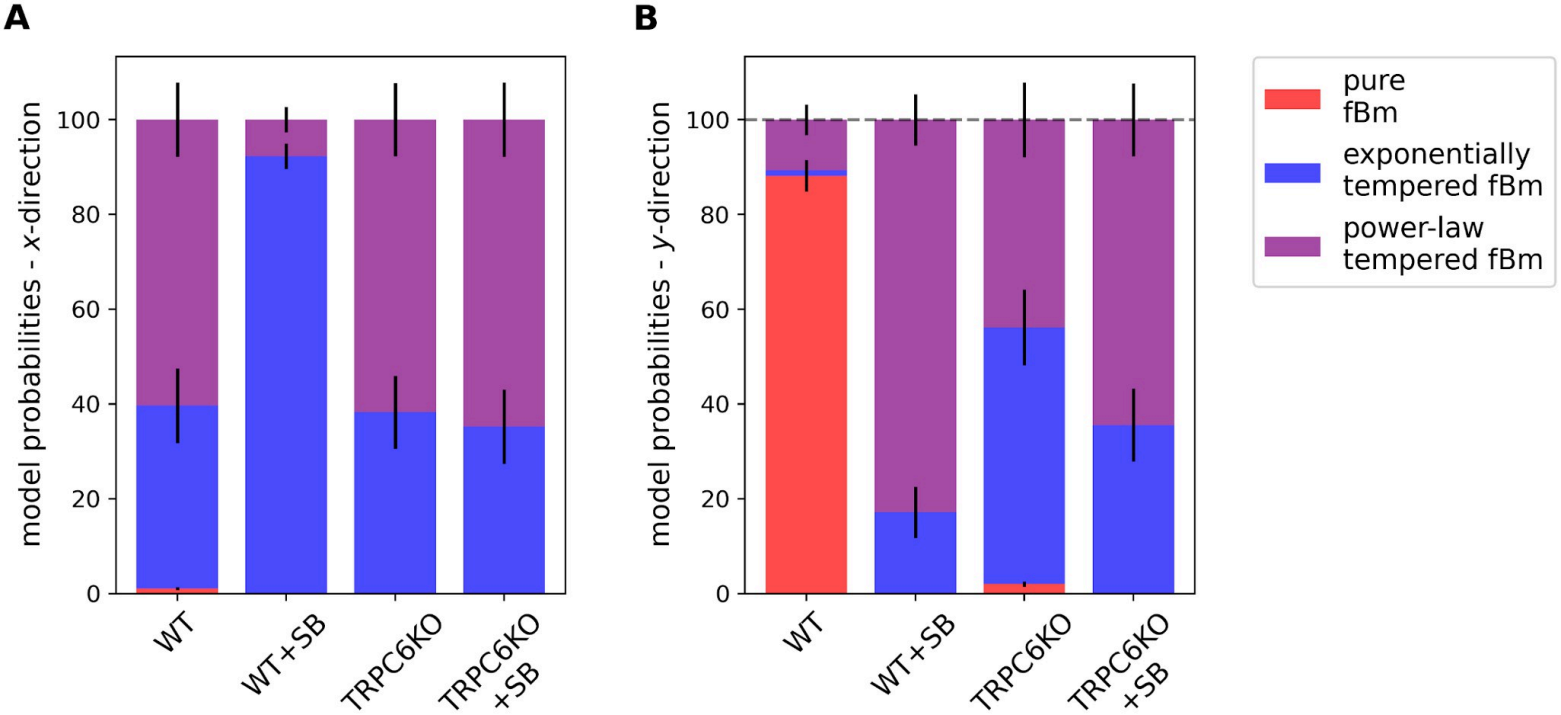
Model probabilities for murine neutrophils. The model probabilities for the analyzed models A—C are color coded and shown with respect to (A) *x*- and (B) *y*-direction (probabilities of model D are too small to be visualized). Wildtype neutrophils follow pure fractional Brownian motion parallel to the chemotactic gradient, whereas the dynamics of neutrophils under all other conditions is either exponentially or power-law tempered.

In the first step we compared the four different models applied to all experimental data. Fig 4 summarizes the model probabilities for each experimental condition. We applied the models separately for *x*- and *y*-coordinates. It is clearly evident that movement of WT neutrophils along the chemotactic gradient (*y*-direction) is best modeled by pure fractional Brownian motion (model A). Whenever directional movement is absent (*x*-direction; Fig 4A) or impaired (*y*-direction of WT+SB, TRPC6KO and TRPC6KO+SB; Fig 4B), models with tempering are favored (model B and C). The necessity of introducing tempered models indicates that the extent of the temporal memory depends on the orientation with respect to the chemotactic gradient (*x* versus *y*) and on CXCR2 signaling and/or TRPC6 channels. Model probabilities of the Ornstein-Uhlenbeck process of model D were extremely low (< 6.510^−51^% for all experimental conditions) and therefore could not be made visible in Fig 4. Therefore, we omitted the Ornstein-Uhlenbeck process of model D from the further analysis. Nevertheless, the analysis revealed the time-scale 1/*γ* ∼ 0.1 … 0.2 min for all four experimental conditions. This indicates that a ballistic phase of migrating cells is hardly present and thus confirms the use of the overdamped modeling approach.

#### Quantitative analysis of model parameters

**Fig 5:**
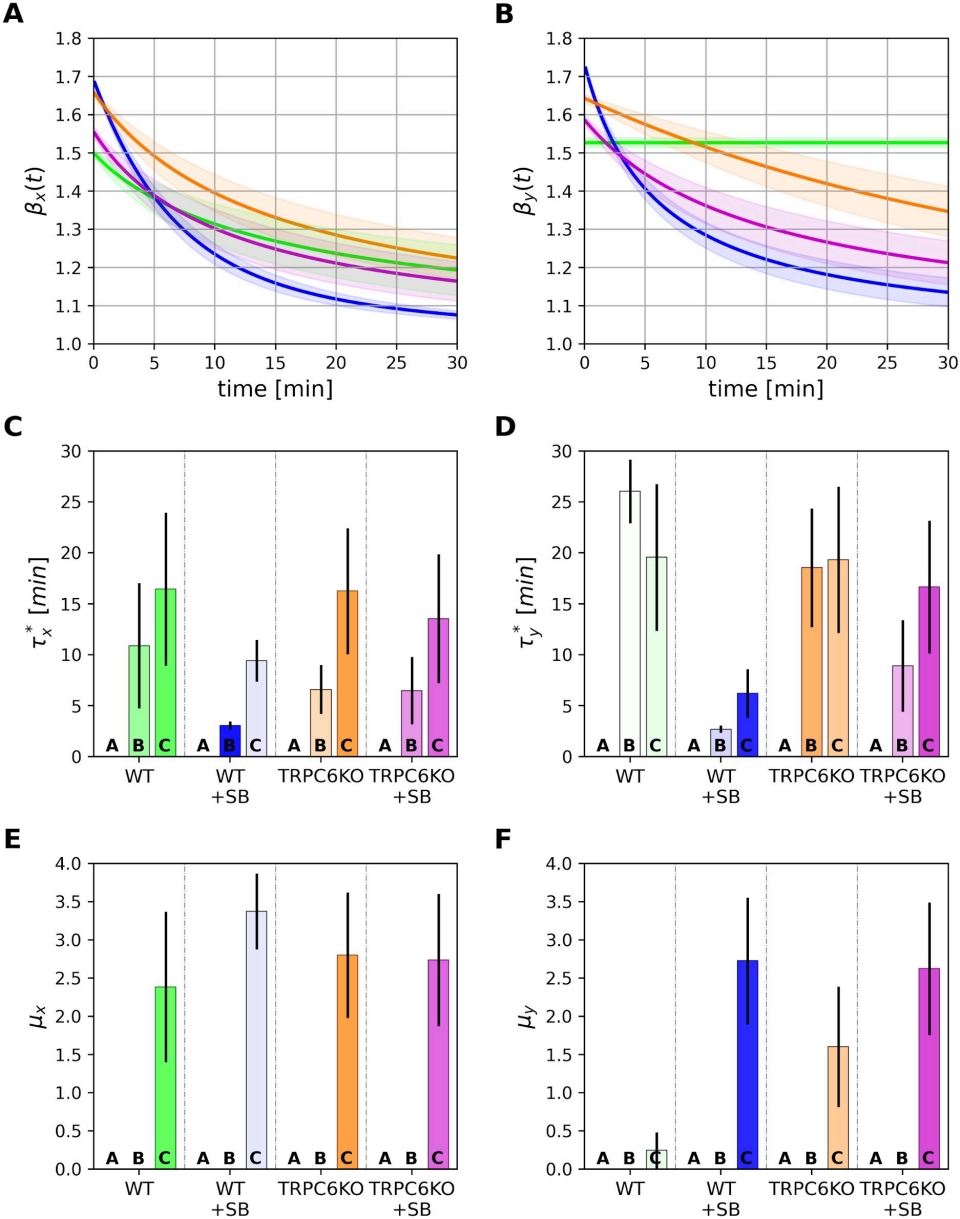
Tempering behavior of murine neutrophils. (A) and (B) show the temporal behavior of the logarithmic derivative *β*(*t*) of the mean squared displacement perpendicular (A) and parallel (B) to the direction of chemotaxis. The time-scale τ* of tempering is compared in (C) and (D) for the four different cell groups (τ* = ∞ for model A, pure fBm). (E) and (F) show the exponent *μ* of power-law tempering of model C. The color intensities of the parameter bars are proportional to the corresponding model probabilities given in Fig 4.

To obtain an overview of the consequences of tempering we first analyzed the logarithmic derivative of the mean squared displacement *β*_*x*|*y*_(*t*) = *d* ln *msd*_*x*|*y*_(*t*)/*d* ln *t* (Fig 5A and 5B) perpendicular and parallel to the direction of the chemotactic gradient. Fig 5B illustrates that only WT neutrophils move with an untempered superdiffusive behavior with a constant *β*_*y*_ ∼ 1.55 during the entire observation period. The parameter *β*_*y*_(*t*) of all other groups decays. The decay is more gradual in TRPC6KO neutrophils and stronger upon CXCR2 blockade. In contrast, the movement vertically to the chemotactic gradient (*x*-direction) is tempered for all four experimental groups in accordance with models B and C (Fig 5A).

A more quantitative analysis of the tempering behavior is obtained by considering the model parameters τ* (tempering time; model B/C) and *μ* (power-law tempering coefficient; model C). Numerical estimates and uncertainties of these parameters for all three models are plotted in Fig 5C–5F. The color intensities of the parameter bars are proportional to the corresponding model probabilities shown in Fig 4. The fact that model A (pure fractional Brownian motion) is favored for WT neutrophils is reflected by an infinite tempering time τ*. TRPC6KO neutrophils have a tempering time in the order of τ* ∼ 20 min. It is further decreased in the presence of the CXCR2 blocker.

The parameter *μ* describes the strength of power-law tempering in model C. Values of *μ* are between 1.5 and 3.0 except for WT neutrophils. Thus, the parameter *μ* is in the range of *μ* > 2*H* − 1 for WT+SB, TRPC6KO and TRPC6KO+SB groups. This indicates so-called strong power-law tempering [34]. Thus, TRPC6KO neutrophils and neutrophils with inhibited CXCR2 receptor perform normal diffusion for long times.

**Fig 6:**
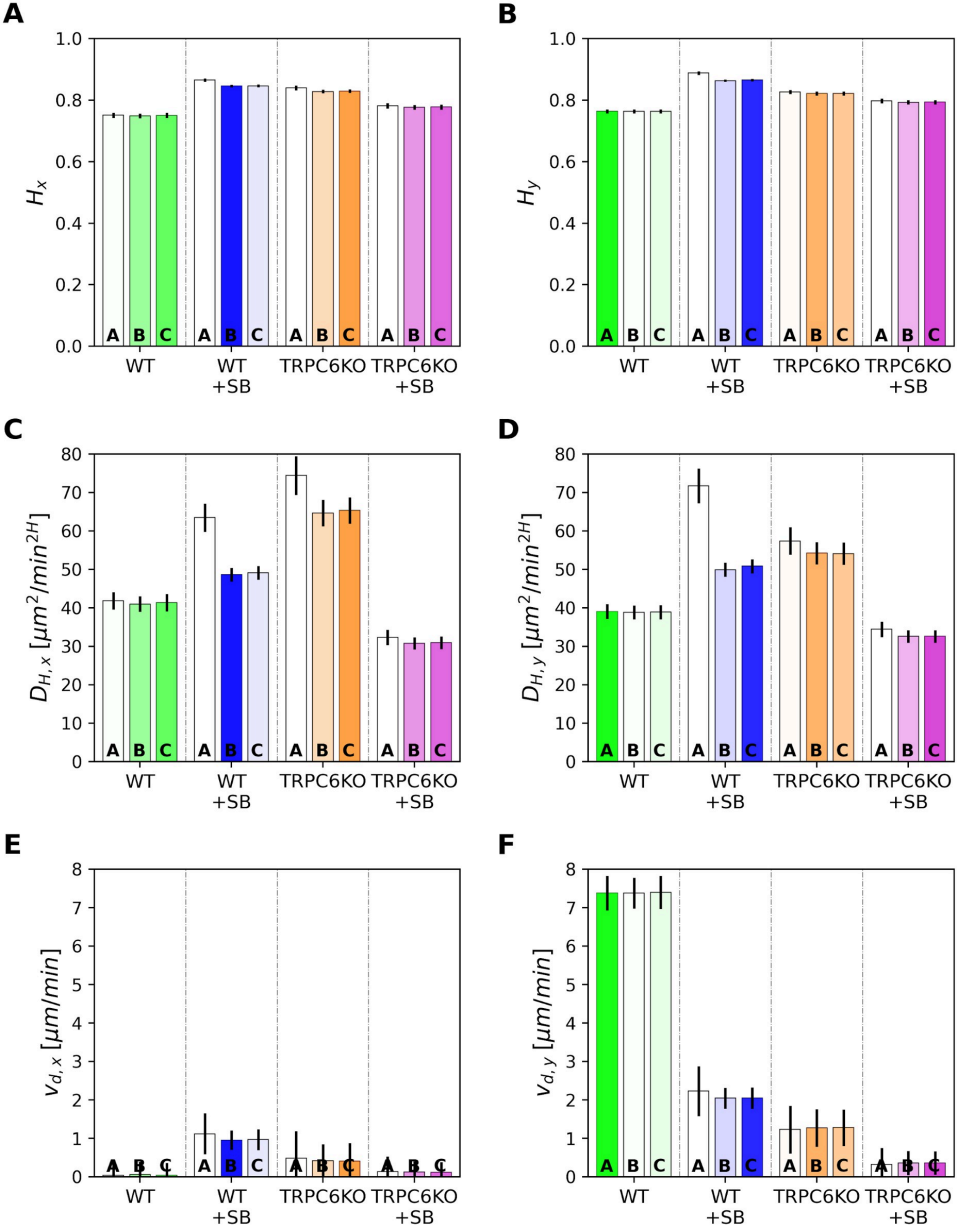
Comparison of model parameters for murine neutrophils. Parameters are plotted in *x*- and *y*-direction together with their standard deviation for the three analyzed models: A—fractional Brownian motion (fBm), B—fBm with exponential tempering, and C—fBm with power-law tempering. The color intensities of the parameter bars are proportional to the corresponding model probabilities in Fig 4. (A) and (B) Hurst exponent *H*, (C) and (D) generalized diffusion coefficient, (E) and (F) absolute value of drift velocity *v*_*d*_.

All three models contain the parameters *H* (Hurst coefficient), *D*_*H*_ (diffusion constant) and *v*_*d*_ (drift velocity) as shown in Fig 6. First, we consider the model parameters for movement in the *y*-direction in Fig 6B, 6D and 6F. Modeling recapitulates that drift velocity of neutrophils moving in a CXCL1 gradient strongly depends on CXCR2 signaling and the presence of TRPC6 channels. It is almost zero when the receptor is inhibited and / or the channel is absent. Model parameters *v*_*d*_ are almost independent of the applied model A—C. The Hurst coefficient of WT neutrophils (Fig 6B) amounts to 0.76±0.01 for the preferred model A indicating a superdiffusive behavior over the entire observation interval. The Hurst coefficients of all other experimental groups are higher. However, superdiffusive dynamics for these cells is tempered as indicated by the preferred models B and C. Thus, superdiffusive behavior of WT+SB, TRPC6KO and TRPCKO+SB neutrophils is rapidly attenuated with time (see Fig 5). The generalized diffusion coefficient *D*_*H*_ (Fig 6D) is about 39.01 ± 1.89 μm^2^/min for WT neutrophils. WT+SB and TRPC6KO neutrophils have a higher diffusion coefficient.

In summary, it is notable that parameters *H* and *D*_*H*_ are quite similar in parallel and vertical to the chemotactic gradient (Fig 6). This could represent a common symmetric dynamics of the cells for small times. Our results also demonstrate that long term behavior is predominately determined by the degree of tempering (parameters τ* and *μ*). Asymmetric tempering is stronger vertical to the direction of chemotaxis. Less tempering in parallel to the chemotactic gradient may favor directed movement which is assessed by parameter *v*_*d*_.

### Behavior of zebrafish neutrophils

**Fig 7:**
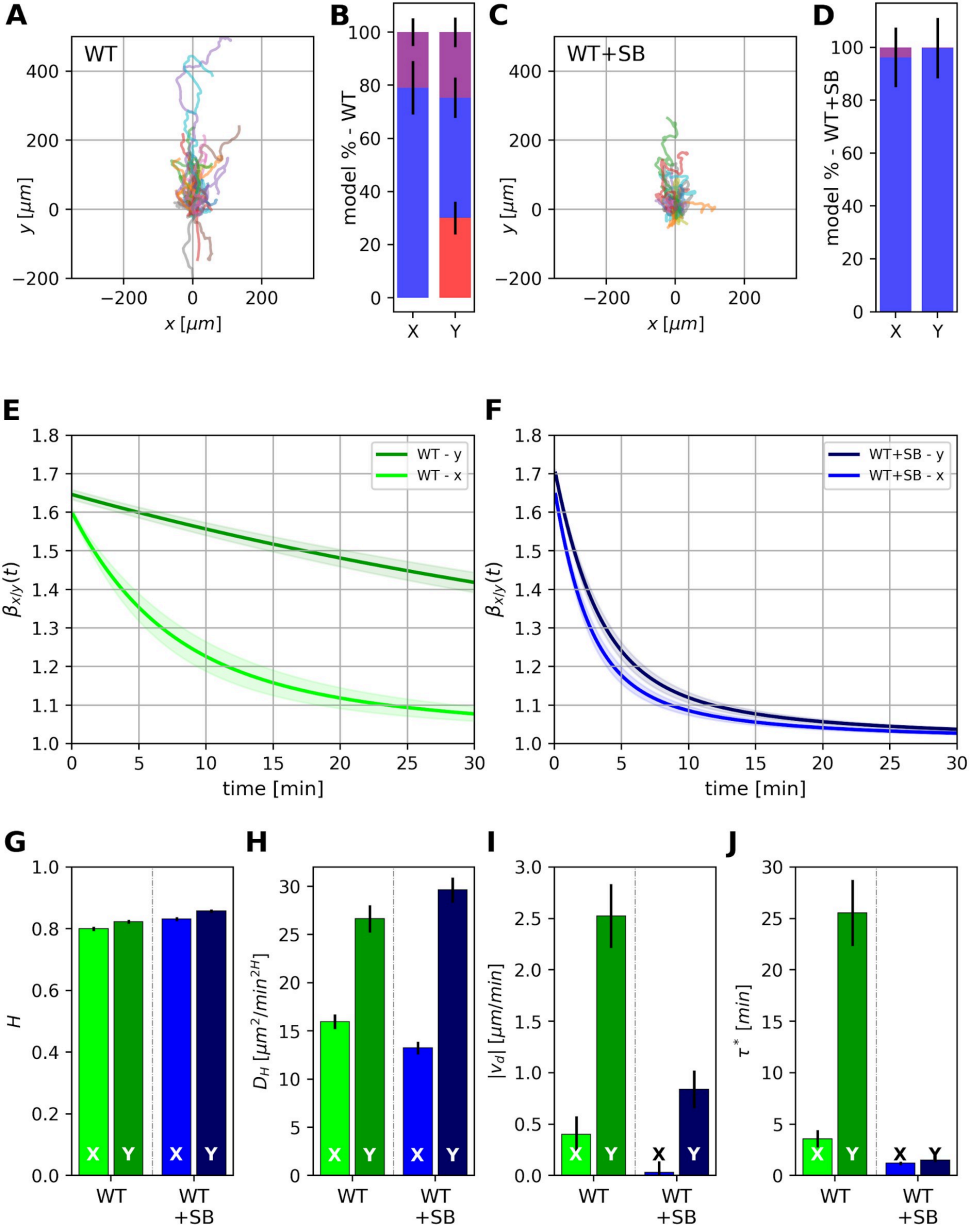
Model parameters for the dynamics of neutrophils in zebrafish larvae. (A) and (C) give an overview of all different trajectories of neutrophils moving in zebrafish larvae. Paths of neutrophils have different lengths. Neutrophils treated with blocker SB225002 (marked as WT+SB) have a reduced drift (C) compared to untreated cells (A). Model probabilities for WT neutrophils (B) and for the WT+SB group favor the exponentially tempered model under all directions and conditions (color coding as in Fig 4). (E) and (F) show the temporal behavior of the logarithmic derivative *β*(*t*) of the mean squared displacement for the two cell groups. WT+SB neutrophils are tempered much more strongly than WT neutrophils. The movement of WT neutrophils in parallel to the direction of chemotaxis (*y*-direction) is only weakly tempered. (G)—(J) show the values of model parameter for fBm with exponential tempering: (G) Hurst exponent, (H) diffusion coefficient *D*_*H*_, (I) absolute value of drift *v*_*d*_, and (J) tempering time τ*.

So far we have analyzed the chemotactic behavior of murine neutrophils in vitro. In the next step we wanted to ensure that these results are also relevant for the in vivo situation. Therefore, we monitored directed movement of neutrophils in a zebrafish model. Neutrophil movement was induced by tail transection and monitored for up to 2 hours. Experiments delivered *N* = 108 and *N* = 106 trajectories with different lengths for control (WT) and SB225002-treated zebrafish (WT+SB), respectively. Fig 7 provides an overview of the trajectories obtained under both conditions (Fig 7A and 7C) and the resulting model parameters (Fig 7E–7J). It is evident that the model-based analysis of the trajectories of zebrafish neutrophils yields qualitatively almost the same results as described above for murine neutrophils (Figs 5 and 6).

Model probabilities for WT neutrophils (Fig 7B) and for the WT+SB group (Fig 7D) are clearly in favor of model B (fractional Brownian motion with exponential tempering). Only for WT neutrophils moving in the direction of chemotaxis, model A and model C show similar, but slightly smaller probabilities than model B. The effect of tempering is illustrated in Fig 7E and 7F by the decay of the logarithmic derivative *β*(*t*) of the mean squared displacement. Movements of WT neutrophils in *y*-direction are only weakly tempered so that their behavior almost fits to the pure fBm of model A. In contrast, tempering vertical to the chemotactic gradient (*x*-direction) is much stronger. As shown in Fig 7F, WT+SB neutrophils are tempered more strongly in both directions by the blockade of the CXCR2 receptor. Finally, parameters for neutrophils moving in zebrafish embryos show Hurst exponents *H* ∼ 0.80 − 0.86 (Fig 7G). The generalized diffusion coefficient *D*_*H*_ is increased in parallel to the direction of chemotaxis for WT and WT+SB neutrophils (Fig 7H). This is most likely due to the anatomical structure of the tail fin of the zebrafish embryo [37]. It is characterized by rays that are aligned in parallel towards the the tip of the tail fin. This also applies to collagen fibers of the extracellular matrix and blood vessels [38]. Thus, these structural cues are in parallel to the direction of the chemotactic gradient which is generated by tail transection. They are relevant directional cues because the topography of the extracellular environment largely influences immune cell migration [39]. In addition to the increased diffusion coefficient, there is a clear directed drift velocity *v*_*d*_ ∼ 2.5 μm/min, which is reduced to *v*_*d*_ ∼ 0.8 μm/min by the blockade of the CXCR2 receptor (Fig 7I). Finally, tempering time τ* is around ∼ 25 min for WT cells in *y*-direction. This time is larger than than mean observation time of cells which is around ∼ 17.1 min and 19.5 min for WT and WT+SB, respectively.

### Model-based simulation

Using the model parameters summarized in Figs 5 and 6 we simulated a large number of trajectories (100000) for each of our experimental conditions. The simulations were performed for the direction of chemotaxis (*y*-direction) using the corresponding covariance in Eqs 8–10 and parameters of the most probable model (Fig 4) for each experimental condition (see Material and methods for simulations based on the covariance function). I.e. model A with the corresponding WT-parameters were applied to simulate WT-conditions. Analogously, model C with the WT+SB parameters, model B with TRPC6KO parameters, and model C with TRPC6KO+SB parameters were used to simulate paths for WT+SB, TRPC6KO, and TRPC6KO+SB neutrophils, respectively.

**Fig 8:**
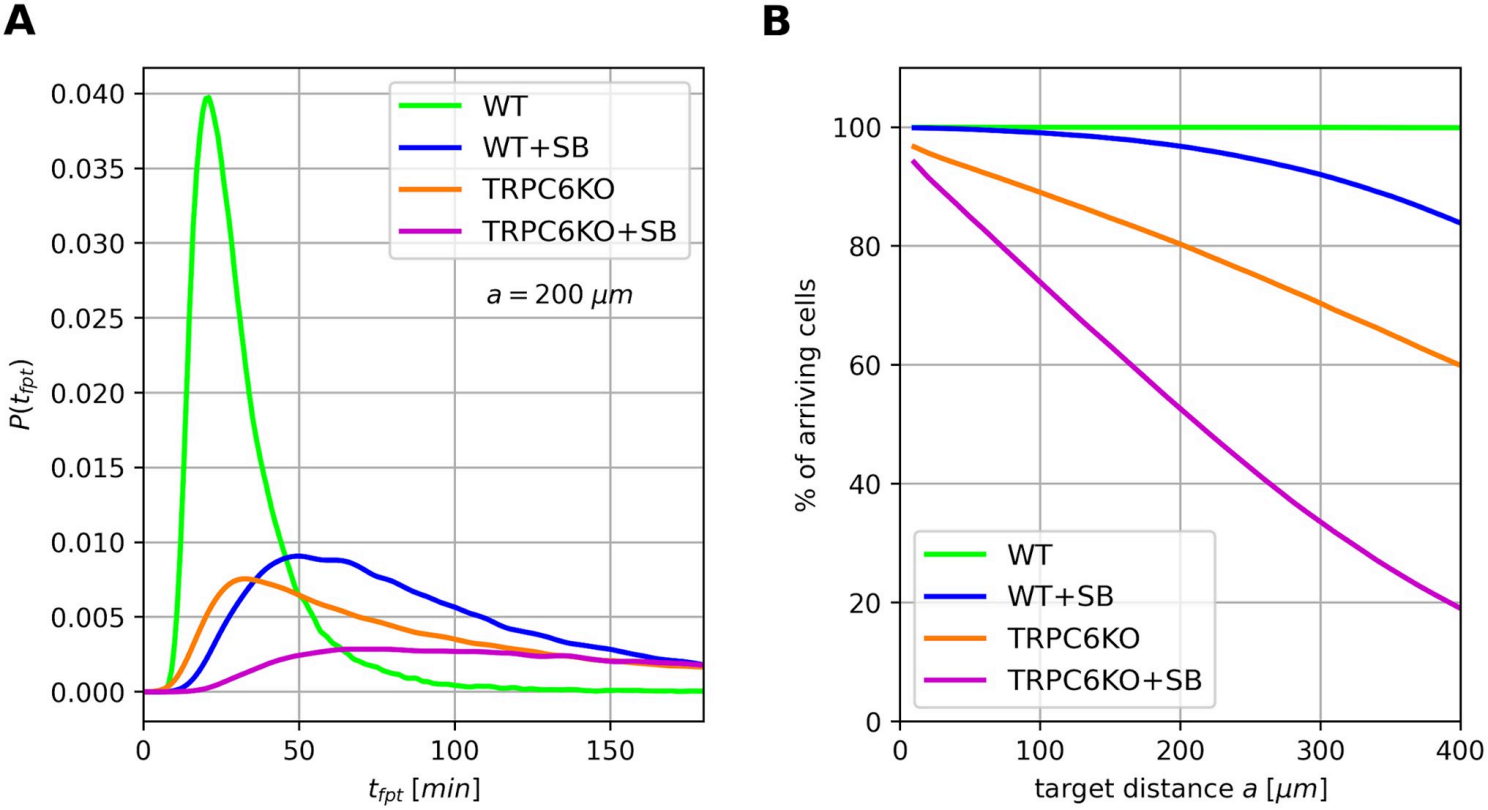
Comparison of arriving probabilities by simulations. (A) Probability distributions of first passage times for simulated cells at a distance of 200 *μm*. Model parameters calculated for the respective experimental conditions were used for simulations as given in Figs 5 and 6. There is a surge of WT neutrophils reaching their target after 20 min, whereas all other cell types show a delayed arrival. (B) Percentage of cells arriving at the target that is located at a distance *a* from the starting points of the cell ensemble. All simulated wildtype cells arrive at the target within 5 h, whereas CXCR2 inhibition and/or TRPC6 ablation markedly reduce the number of cells arriving at their target.

We used these simulations to make predictions about the long-term search behavior of neutrophils and evaluated their search efficiency under the assumption that model parameters remain constant over the considered time period. The search behavior is quantified by the so-called first passage time *t*_*fpt*_ describing the duration neutrophils need to pass a target for the first time [40]. Fig 8A shows the probability of first passage times *P*(*t*_*fpt*_) for neutrophils reaching a target at distance *a* = 200 μm apart from their starting point. This corresponds to the distance neutrophils are physiologically covering within the interstitium during swarming [41]. Wildtype neutrophils come in rapidly, whereas neutrophils from all other groups (TRPC6-knockout and/or CXCR2 blockade) arrive in a trickle. This is consistent with the kinetics of neutrophil swarming which begins after a lag phase of ∼ 15 min and which is impaired in CXCR2^−/−^ neutrophils [42]. In Fig 8B the percentage of arriving cells after a time period of 5 h is plotted as a function of the target distance *a*. All simulated wildtype neutrophils reach the target within the observation period, while TRPC6-knock-out and CXCR2 blockade strongly reduce the number of arriving cells. Notably, the simulation results with parameters for TRPC6^−/−^ neutrophils correspond quite well to our previous data on neutrophil recruitment in a peritonitis model [11]. Here, the number of recruited TRPC6^−/−^ neutrophils is reduced to 60–70% of that in wildtype animals. The distance of capillaries to the peritonial surface is in the order of 100–400 μm [43]. Hence, such simulations could also be employed for planning the observation time interval e.g. when using end-point measurements of neutrophil emigration.

## Discussion

We developed three stochastic models based on anomalous dynamics and tempering mechanisms and applied them together with the Ornstein-Uhlenbeck process to analyze neutrophil chemotaxis under different experimental conditions. Using a gradient of CXCL1 we examined chemotacting murine neutrophils under control conditions, in the presence of a CXCR2 blocker and in the absence of TRPC6 channels. In addition, murine neutrophils chemotacting in vitro were compared with the chemotactic behavior of neutrophils in zebrafish embryos. Our model-based approach reveals clear differences between the experimental conditions that were not as evident from previous conventional analysis [11]. CXCR2 inhibition has a more profound effect on the dynamics of chemotaxis than TRPC6-knockout suggesting that (i) TRPC6 channels are positioned downstream of the receptor and that (ii) CXCR2 receptors trigger other, TRPC6-independent intracellular signaling cascades. If all CXCR2 signaling had to pass via TRPC6 channels, its deletion would have elicited the identical phenotype. Yet, tempering and drift velocity are more dependent on CXCR2 receptors than on TRPC6 channels.

One of the key result is the finding that there is an asymmetry of anomalous migration dynamics in the direction of the chemotactic gradient and in transverse direction. Importantly, this asymmetric anomaly is also observed in vivo in the zebrafish embryo. This asymmetry appears to be a prerequisite for efficient chemotaxis. To the best of our knowledge this is the first description of asymmetric anomalous dynamics during chemotaxis in an intact organism as revealed by the strong and weak tempering behavior (see Fig 7E) in *x*- and *y*-direction, respectively. In an in vivo model of *Toxoplasma gondii* infection of the brain, migration of CD8+ T cells within the brain was described as a generalized Lévy walk, which was accelerated in a chemokinetic way by CXCL10 [16]. Thus, migration of CD8+ T cells appears to harbour anomalous properties as well [16]. Our analysis goes one step further in that we are analyzing chemotaxis and show the asymmetry and tempering of anomalous behavior parallel and perpendicular to the direction of the chemotactic gradient.

Moreover, we found that the anomalous properties of the neutrophils can be modulated by distinct molecular mechanisms. We discovered that the degree of anomaly and of its asymmetric tempering strongly depend on CXCR2 and to a lesser extent on TRPC6 channels. Accordingly, the parameter *β*(*t*) decays faster in blocker treated neutrophils than in TRPC6KO cells. As stated above, this also applies to neutrophils in zebrafish larvae. In our view it is likely that the higher degree of memory (corresponding to less tempering) in the direction of the chemotactic gradient favors the biased formation of pseudopods [15] or the biased activity of an excitable network and polarization underlying the polarized local excitation and global inhibition (LEGI-BEN) model [13]. Along these lines our analysis indicates that CXCR2 receptors and TRPC6 channels have a major impact on the bias of the excitable network as evidenced by the markedly reduced drift velocity when these membrane proteins are inhibited or absent. In contrast, TRPC6 channels have only a smaller effect on tempering and its asymmetry which could be reflected by the ‘polarization’ term in this model [26]. The combined effect, however, is sufficient to account for poor chemotaxis under both conditions. In addition, TRPC6 channels may be involved in the sensitivity of the gradient detection which depends on the polarization of the cells. In contrast, the CXCR2 inhibition disturbs drift velocity, tempering and asymmetry indicating that bias and polarization are strongly impaired.

Thus, by monitoring the migratory behavior on a ‘macroscopic’ level and performing a model-based analysis of the cell trajectories, we are able to obtain quantitative information on the molecular mechanisms underlying neutrophil chemotaxis. The effects of CXCR2 blockade or TRPC6-knockout may serve as proof-of-principle in this respect. As proposed earlier [44], CXCR2 receptors and TRPC6 channels seem to be signaling modules that transduce the ‘bias’ of newly formed pseudopodia [15] or of the excitable network within the lamellipodium [13]. Future studies will have to show whether this is linked to a polarized distribution of TRPC6 channel proteins or to localized activity of TRPC6 channels at the front of chemotaxing neutrophils. Then the channels could initiate local Ca^2+^ signaling domains as described earlier [6, 45–48]. The latter possibility is supported by observations made in fibroblasts [49] where DAG [50], one of the main physiological activators of TRPC6 channels [51], was found to be enriched at the cell front.

The efficiency of the chemotaxis of neutrophils is related to the strength of tempering of the anomalous behavior: We observe optimal chemotaxis of WT neutrophils where tempering is absent in the direction of chemotaxis and present perpendicular to the chemotactic gradient. In this case anomality and temporal memory are maintained for the entire period of observation. In addition, the resulting drift velocity *v*_*d*_ has a high value. Conversely, the absence of asymmetry and increased tempering in the direction of the gradient for TRPC6KO neutrophils and CXCR2 blockade is accompanied by an impaired chemotaxis, i.e. a reduced drift velocity *v*_*d*_. Then even the seemingly small additive effect of TRPC6 channel deletion and CXCR2 blockade [11] (see also Fig 2) translate to marked differences in search efficiency. For small times, the higher generalized diffusion coefficient *D*_*H*_ of TRPC6KO versus WT+SB neutrophils favors the TRPC6KO first passage times. However, for longer times the higher drift velocity of WT+SB neutrophils gets dominant resulting in a higher percentage of arriving cells at a given target distance. These in silico results may have important implications for pathologies associated with CXCR2 signaling. One such example is the CXCR2-dependent recruitment of neutrophilic myeloid derived suppressor cells to cancers such as pancreatic ductal adenocarcinoma [52], breast cancer [53] or lung cancer [54]. In addition, our findings are also relevant for interpreting search behavior and optimal search strategies of anomalous processes [16, 55–57].

## Material and methods

### Ethics statement

Experimental data of murine neutrophils are taken from Lindemann et al. [11] without further experiments (data from [11] were conducted with the permission 84–02.05.50.15.010 of the Landesamt für Natur, Umwelt und Verbraucherschutz Nordrhein-Westfalen, Germany). Adult and larval zebrafish were maintained according to protocols approved by the University of Wisconsin-Madison Institutional Animal Care and Use Committee [27].

### Cells—Murine neutrophils

We further analyzed data sets of neutrophils [11] isolated from wildtype and TRPC6^−/−^ mice migrating in chemotactic gradients of CXCL1 (keratinocyte-derived cytokine; CXCL1). Chemotaxis of both cell types was monitored in the absence and presence of the CXCR2 blocker SB225002 (500 nM). Chemotaxis experiments were performed in chemotaxis chambers (ibidi, Martinsried, Germany). Cells were seeded within a three-dimensional collagen I matrix (2.1 mg/ml; pH 7.4; BD, Heidelberg, Germany). Chemotaxis was recorded over a period of *T* = 30 min with a time sampling interval of *dt* = 5 s. Trajectories were obtained by tracking the movement of the cell center resulting in 361 *x*-*y*-positions for each cell path [11]. *N* = 60 and *N* = 100 tracks were used for the analysis of wild-type neutrophils and TRPC6^−/−^ cells, respectively.

### In vivo—Zebrafish neutrophils

In vivo experiments in previously described Tg (mpx:Dendra2) zebrafish larvae expressing green fluorescent neutrophils were performed to quantify neutrophil recruitment to wounds [27]. 3 dpf Tg (mpx:Dendra2) zebrafish larvae were pre-treated for 30 minutes with 5 *μ*M of CXCR2 inhibitor SB225002 or with DMSO as control. Larvae were then tail transected and mounted in 1% agarose containing 5 *μ*M SB225002 or DMSO. Neutrophil recruitment to the wound was recorded with a mean sampling interval of *dt* = 17 s up to 2 h with a laser scanning confocal microscope (FluoView FV1000, Olympus). The analysis of the video sequences and extraction of cell paths was performed with self-developed image processing software (based on python and the openCV library).

### Data analysis

Quantitative data analysis and calculation of parameters were performed with extensions of programs developed by ourselves [23]. Cell trajectories were used to calculate the first and second moments. We analyze time-averages of single trajectories *x*_*k*_(*t*) of cell *k* as a function of time *t* in the interval [0, *T*]. These are defined as

$$\begin{array}{ccc} \bar{x_{k}\left(t\right)} & = & \frac{1}{T-t}\int_{0}^{T-t}\;dt^{\prime }\;\left[x_{k}\left(t+t^{\prime }\right)-x_{k}\left(t^{\prime }\right)\right]\;, \end{array}$$ (16)

$$\begin{array}{ccc} \bar{x_{k}^{2}\left(t\right)} & = & \frac{1}{T-t}\int_{0}^{T-t}\;dt^{\prime }\;{\left[x_{k}\left(t+t^{\prime }\right)-x_{k}\left(t^{\prime }\right)\right]}^{2}\;, \end{array}$$ (17)

where the over-line indicates the time-average. The time-ensemble-average is given by the mean over all *N* cell paths *k* = 1 … *N*:

$$\begin{array}{ccc} \left\langle x(t)\right\rangle & = & \frac{1}{N}\sum_{k=1}^{N}\bar{x_{k}\left(t\right)}\;, \end{array}$$ (18)

$$\begin{array}{ccc} \left\langle x^{2}\left(t\right)\right\rangle & = & \frac{1}{N}\sum_{k=1}^{N}\bar{x_{k}^{2}\left(t\right)}\;. \end{array}$$ (19)

The definitions for the *y*-direction apply analogously. The second moment is synonymously called mean squared displacement in the present work, i.e. *msd*_*x*_(*t*) = 〈*x*^2^(*t*)〉 and *msd*_*y*_(*t*) = 〈*y*^2^(*t*)〉. It should be noted here that the mean squared displacement is defined as non-central moment. We do not use central moments, because central moments in combination with time-ensemble averages and fractional dynamics cause several problems for systems with drift [58, 59]. In addition, our model analysis is based on the position of cells and not on the moments of positions. The velocity of migrating cells in *x* and *y*-direction *v*_*x*|*y*_(*t*) was calculated from trajectories as the difference quotient of two cell positions at times *t* + Δ and *t*.

$$\begin{array}{c} v_{x}\left(t\right)=\frac{x(t+\Delta )-x(t)}{\Delta },\;\;\;v_{y}\left(t\right)=\frac{y(t+\Delta )-y(t)}{\Delta }\;. \end{array}$$ (20)

For Δ the corresponding sampling interval of the experiments was applied. These velocities were used to calculate the autocorrelation function of velocities in *x*- and *y*-direction. Analogously to the time-average and time-ensemble-average definitions for the moments in Eqs 16–19 velocity autocorrelations are defined as

$$\begin{array}{ccc} \bar{{\text{v}}_{corr,x,k}\left(t\right)} & = & \;\frac{1}{T-t}\int_{0}^{T-t}\;dt^{\prime }\;\left[v_{x,k}\left(t+t^{\prime }\right)\;v_{x,k}\left(t^{\prime }\right)\right]\;, \end{array}$$ (21)

$$\begin{array}{ccc} \left\langle {\text{v}}_{corr,x}\left(t\right)\right\rangle & = & \frac{1}{N}\sum_{k=1}^{N}\bar{{\text{v}}_{corr,x,k}\left(t\right)}\;. \end{array}$$ (22)

These definitions apply analogously to the *y*-direction. Accordingly, the time- and ensemble-averaged velocity cross-correlation function between velocities in *x*- and *y*-direction can be defined as

$$\begin{array}{ccc} \bar{{\text{v}}_{cross,xy,k}\left(t\right)} & = & \;\frac{1}{T-t}\int_{0}^{T-t}\;dt^{\prime }\;\left[v_{x,k}\left(t+t^{\prime }\right)\;v_{y,k}\left(t^{\prime }\right)\right]\;, \end{array}$$ (23)

$$\begin{array}{ccc} \left\langle {\text{v}}_{cross,xy}\left(t\right)\right\rangle & = & \frac{1}{N}\sum_{k=1}^{N}\bar{{\text{v}}_{cross,xy,k}\left(t\right)}\; \end{array}$$ (24)

to study possible couplings of velocities between different directions.

### Bayesian inference of model parameters based on the covariance of the stochastic process

Gaussian stochastic processes as e.g. for positions *x*(*t*) are completely determined by their first moment *μ*(*t*) and covariance ***C***:

$$\begin{array}{c} \mu \left(t\right)=\left\langle x\left(t\right)\right\rangle ,\;\;\;C\left(t,t^{\prime }\right)=\left\langle [x(t)-\mu (t)][x\left(t^{\prime }\right)-\mu \left(t^{\prime }\right)]\right\rangle \end{array}$$ (25)

An experimental sampling of a single trajectory of this process delivers *M* data points ***d*** = {*d*(*t*_1_), *d*(*t*_2_) … *d*(*t*_*M*_)} at times *t*_*i*_ with *i* = 1 … *M*. The resulting likelihood $L_{s}$ for one cell path is given by [60–63]

$$\begin{array}{c} L_{s}\left(d|C,\theta \right)=\frac{\text{exp}\{-\frac{1}{2}\;(d\;-\;\mu \left(t\right){)}^{⊺}\;C^{-1}\;(d\;-\;\mu \left(t\right))\}}{{\left(2\pi \right)}^{M/2}\;{\left[\text{det}\;C\right]}^{1/2}}\;. \end{array}$$ (26)

The mean drift ***μ*** and the covariance ***C*** may depend on model parameters ***θ*** with

$$\theta =\{\begin{array}{ll} \left(H,D_{H},v_{d}\right) & \text{for}\;\text{model}\;\text{A}\\ \left(H,D_{H},v_{d},\tau^{*}\right) & \text{for}\;\text{model}\;\text{B}\\ \left(H,D_{H},v_{d},\tau^{*},\mu \right) & \text{for}\;\text{model}\;\text{C}\\ \left(\gamma ,v_{m}^{2},v_{d}\right) & \text{for}\;\text{model}\;\text{D} \end{array}$$ (27)

(compare with Eqs 6–10). The likelihood of a cell ensemble consisting of *N* paths with data ***d***_*k*_ (*k* = 1 … *N*) is just the product of the corresponding individual likelihoods:

$$\begin{array}{c} L\left(d|C,\theta \right)=\prod_{k=1}^{N}\;L_{s}\left(d_{k}|C,\theta \right)\;. \end{array}$$ (28)

Eq 28 can now be applied within the context of Bayesian data analysis [35, 36, 63] to estimate parameters of Gaussian stochastic models defined by their first moment and covariance in the light of the available experimental data. According to the Bayesian theorem, the posterior distribution of parameters *p*(***θ***|***d***) is given as

$$\begin{array}{c} p\left(\theta |d\right)=\frac{1}{Z}\;L\left(d|C,\theta \right)\;p\left(\theta \right)\;. \end{array}$$ (29)

L(d|C(θ) represents the likelihood as defined in Eq 28. The so-called prior distribution *p*(***θ***) contains all information about the model parameters without any relation to the current experimental data set. *Z* is the so-called evidence: It normalizes the posterior distribution and gives information about the model probability. We apply the nested sampling algorithm [64, 65] as a numerical technique to obtain the marginalized posterior distributions of parameters and model probabilities. Mean parameter values and their uncertainties are contained in the posterior of parameters *p*(***θ***|***d***). They are obtained formally from the calculation of moment integrals over the posterior with respect to the parameters *θ*_1_ … *θ*_*M*_ (for *M* parameters):

$$\begin{array}{c} \left\langle \theta_{k}^{n}\right\rangle =\frac{1}{Z}\;\int \;\prod_{j\neq k}^{M}d\theta_{j}\;\theta_{k}^{n}\;L\left(d|\theta_{1}\ldots \theta_{M}\right)\;p\left(\theta_{1}\ldots \theta_{M}\right)\;. \end{array}$$ (30)

The mean 〈*θ*_*k*_〉 of the parameter *θ*_*k*_ is obtained for *n* = 1, its standard deviation with *n* = 2 by

$$\begin{array}{c} \delta \theta_{k}=\sqrt{\left\langle \theta_{k}^{2}\right\rangle -{\left\langle \theta_{k}\right\rangle }^{2}}\;. \end{array}$$ (31)

These values are given as mean values and error bars for all parameters in Figs 5–7. Thereby, we use weak constant priors for *H* ∈ [0.01, 0.99], *v*_*d*_ ∈ [−25, 25] μm/min, τ* ∈ [0, 30] min, *μ* ∈ [0, 4], and an exponential prior ∼ exp(−*D*_*H*_/*D*_*m*_) for *D*_*H*_ with *D*_*m*_ = 50 μm^2^/min^2*H*^ [35, 36]. In this work we apply Eqs 28 and 29 to our experimental paths of neutrophils using the stochastic Gaussian models defined by Eqs 8–10.

The nested sampling algorithm, which is used to calculate the integrals in Eq 30, delivers numerical sampling curves of parameters and evidences until convergence [64, 65]. Each sampling point *i* (*i* = 1 … *N*_*S*_ with *N*_*S*_ being the number of sampling points) receives a normalized weight *w*_*i*_ ($\sum_{i=1}^{N_{S}}\;w_{i}=1$). These weights are used to calculate mean values and uncertainties of a parameter *θ*_*k*_

$$\begin{array}{ccc} \left\langle \theta_{k}\right\rangle & = & \sum_{i=1}^{N_{S}}\;w_{i}\;\theta_{k,i}, \end{array}$$ (32)

$$\begin{array}{ccc} \left\langle {\left[\theta_{k}\right]}^{2}\right\rangle & = & \sum_{i=1}^{N_{S}}\;w_{i}\;{\left[\theta_{k,i}\right]}^{2} \end{array}$$ (33)

where Eq 31 applies analogously to calculate *δθ*_*k*_. In addition, this procedure is also applied to calculate the mean and standard deviations of functions *f*(***θ***) depending on the parameters ***θ***:

$$\begin{array}{c} \left\langle f^{n}\left(\theta_{1}\ldots \theta_{M}\right)\right\rangle =\sum_{i=1}^{N_{S}}\;w_{i}\;f^{n}\left(\theta_{1,i}\ldots \theta_{M,i}\right)\;. \end{array}$$ (34)

This delivers the real mean value and standard deviation of the function *f*, which may be different from just calculating the function *f* for the mean parameters ***θ***. This calculation is applied to obtain the mean logarithmic derivation *β*(*t*) of the msd in Figs 5 and 7.

Model probabilities are obtained from the corresponding evidences *Z* of all considered models A–D. E.g. the probability *P*(*A*) of model A is given by

$$\begin{array}{c} P\left(A\right)=\frac{Z_{A}}{Z_{A}+Z_{B}+Z_{C}+Z_{D}}\;. \end{array}$$ (35)

Thus, individual model probabilities are between 0 … 1. The definition shows that the sum of the model probabilities of all considered models sums up to one. The resulting model probabilities are shown in Figs 4 and 7 as percentages between 0 … 100%.

### Simulations based on the covariance function

Gaussian stochastic processes are completely specified by their mean and covariance. Thus, mean and covariance can be used for simulating trajectories of the corresponding stochastic process. Covariance matrices as given in Eqs 8–10 are real, symmetric and have positive eigenvalues. Thus, the covariance $C\left(t,t^{\prime }\right)=\langle x\left(t\right)x\left(t^{\prime }\right)\rangle$ can be factorized by a Cholesky decomposition [66] $C\left(t,t^{\prime }\right)=A\;A^{T}$ into a product of a lower triangular matrix A with its transpose $A^{T}$. With uncorrelated Gaussian random variables $\eta_{i}∼N\left(0,1\right)$ (*i* = 1…*N*), *N* positions of the corresponding trajectory are given as a matrix product x=Aη with ***x*** = {*x*_1_, *x*_2_, …*x*_*N*_}. This is obvious [67, 68] from the calculation of expectation values as

$$\begin{array}{c} \left\langle x\;x^{T}\right\rangle =\left\langle A\;\eta \;\eta^{T}\;A^{T}\right\rangle =A\;\left\langle \;\eta \;\eta^{T}\right\rangle \;A^{T}=A\;I\;A^{T}=A\;A^{T}\equiv C\left(t,t^{\prime }\right) \end{array}$$ (36)

where $\langle \eta \;\eta^{T}\rangle =I$ with the identity matrix I has been used. Thus, after performing the Cholesky decomposition of the covariance C, sample trajectories of the stochastic process can be generated according to x=Aη. This method can quite simply be applied when covariances are known [69]. As the numerical efficiency of this procedure is low, specialized algorithms have been developed for specific stochastic processes as fractional Brownian motion (see e.g. [67, 69]). However, for purposes of this work the covariance algorithm was sufficient to perform simulations of the suggested anomalous processes.

The python programs for Bayesian analysis including covariance can be found on the zenodo archive (DOI:10.5281/zenodo.6325568).

## Supporting information

S1 Data Excel file with data shown in Fig 1. (XLSX)

S2 Data Excel file with data shown in Fig 2. (XLSX)

S3 Data Excel file with data shown in Fig 3 and additionally including the velocity cross correlations 〈*v*_*cross*,*xy*_(*t*)〉. (XLSX)

S4 Data Excel file with data shown in Fig 4 and additionally including the model probabilities of the Ornstein-Uhlenbeck process (model D). (XLSX)

S5 Data Excel file with data shown in Fig 5. (XLSX)

S6 Data Excel file with data shown in Fig 6. (XLSX)

S7 Data Excel file with data shown in Fig 7. (XLSX)

S8 Data Excel file with data shown in Fig 8. (XLSX)
